# Assessment of Skin and Mucosa at the Equine Oral Commissures to Assess Pathology from Bit Wear: The Oral Commissure Assessment Protocol (OCA) for Analysis and Categorisation of Oral Commissures

**DOI:** 10.3390/ani12050643

**Published:** 2022-03-03

**Authors:** Mette Uldahl, Louise Bundgaard, Jan Dahl, Hilary Mary Clayton

**Affiliations:** 1 Vejle Equine Practice, Fasanvej 12, 7120 Vejle Øst, Denmark; mette@vejlehestepraksis.dk; 2 Department of Veterinary Clinical Sciences, University of Copenhagen, Agrovej 8, 2630 Taastrup, Denmark; lb@sund.ku.dk; 3 Jan Dahl Consult, Østrupvej 89, 4350 Ugerløse, Denmark; jd@lf.dk; 4 Sport Horse Science, 3145 Sandhill Road, Mason, MI 48854, USA

**Keywords:** horse, oral commissure assessment protocol, oral pathology, oral lesions, bit use

## Abstract

**Simple Summary:**

The goal was to implement a protocol to categorize and record pathological findings at the lip commissures. First, the precise anatomical location was defined; second, pigmentation was classified as natural or potentially pathological; and third, pathological lesions were characterized. Associations between the horse’s history and the observed findings were sought. A convenience sample of 206 horses presented for routine dental prophylaxis were sedated; the mouth was held open with a gag, and photographs were taken of the oral commissures and adjacent areas of the lips. The horses were divided in two groups: horses that had never been bitted and horses currently or previously bitted. The owners completed a questionnaire describing the horse’s name, identification number, breed, age, sex, colour, training level, and discipline. The photographs were analysed in a systematic manner, and a score chart was developed to standardize the reporting of lesions. Potentially pathological pigment changes occurred more frequently in horses with a higher level of training (*p* = 0.04) and light-coloured horses (*p* = 0.0004), but were not associated with current bit use or the discipline that the horse participated in (*p* = 0.20). Scars were more frequent in horses competing at a higher level. Only two horses had contusions/erosions, five had ulcers and none showed bleeding; these numbers were too low for statistical analysis.

**Abstract:**

This study addresses the presence and location of natural pigmentation, potentially pathological changes in pigment, interruptions of the natural lining (scars), roughness, and erosions/contusion (bruising) in and around the corners of the lips of 206 horses presented to a veterinarian for routine preventative dental treatment. After sedation, photographs were taken and later evaluated for the presence of lesions. During the photographic analysis, the Oral Commissure Assessment (OCA) protocol was developed to map precisely the areas of skin and mucosa around the corners of the lips, and the presence of lesions was recorded for each area. Potentially pathological pigment changes occurred more frequently in horses with a higher level of training (*p* = 0.04) and in light-coloured horses (*p* = 0.0004), but there was no association with the current use of a bit or the discipline that the horse participated in (*p* = 0.20). Scars occurred more frequently in horses competing at a higher level. Only two horses had contusions or erosions, five had ulcers, and none showed bleeding; these numbers were too low for statistical analysis. Using the OCA protocol provides a detailed method for categorizing and recording lesions in and around the corners of the lips, including natural vs. potential and/or definite pathological character.

## 1. Introduction

Equestrian sports are based on trust and co-operation between a horse and human. The equipment used to facilitate the rider’s control and safety must also be comfortable and humane for the horse. A bit placed within the horse’s inter-dental space has been used for centuries as a means of control [1]. In modern times, bits are manufactured in a variety of shapes and sizes, but their location inside the oral cavity makes it difficult to fully describe their position and action, although fluoroscopic studies have provided some information [2,3]. Lesions associated with the use of bits are more readily seen and described. Since the bit lies in or close to the oral commissures, this is a common site for bit-related lesions.

The most frequently reported type of pathological lesion associated with bit use is oral ulceration [2,3,4,5,6]. Ulcers were also reported in horses that had not been bitted or ridden for 11 months, but these were smaller and less numerous than in ridden horses [3].

Several studies have evaluated horses immediately after different types of competitions for the presence of oral lesions, but direct comparisons across studies are difficult due to different circumstances, such as the use of sedation and a speculum, and the use of different scoring schemes. When horses were examined immediately after dressage and show jumping competitions, 9.2% had lesions around the oral commissures [6]. In that study, horses competing at higher levels showed lesions more frequently but there was no association between the use of a bit or type of bit [6]. In event horses, evaluated after completion of the cross-country phase, 52% had acute oral injuries, and 39% of the lesions were located on the inner aspect of the oral commissures [5]. Horses wearing bits with a thick (>17 mm) or thin (<14 mm) mouthpiece diameter were at greater risk of severe lesions in that study. Trotting horses examined post-racing were described according to criteria established by the authors as a basis for classifying them as no lesions (16%), mild lesions (21%), moderate lesions (43%) or severe lesions (20%) [4]. Racehorses wearing snaffle bits, which are non-leverage bits, were reported to have more severe injuries at the lip commissures and more bone spurs on the mandibles than polo ponies wearing gag bits [2]. In a gag bit, the cheek piece slides through the bit ring and connects directly to the rein so that rein tension raises the mouthpiece into the lip commissures. Prior to the start of a competition, 36% of Icelandic horses had mild oral lesions that appeared to be chronic in nature and were most often located in the buccal mucosa. Severe lesions were found in 8% of horses, most of which were buccal ulcers. When re-evaluated after the preliminary competitions, most of the new lesions were located on the bars in association with using a curb bit [7] in which the cheekpiece attaches above the mouthpiece and the rein attaches below, so the rein tension has a leverage effect.

The correlation between dental findings and ulcers was studied. The presence of hooks on the rostral aspect of the upper second premolar teeth (#106 and/or #206) or sharp enamel points on the buccal aspect of the rostral one third of the left and right upper dental arcades was not related to ulcers at the lip commissures in that study [8].

Apart from ulcers, other markers potentially developing from the use of the bit are a subject of debate. Empirically, depigmentation and scarring in the area of the bit have been seen as markers of previous bit wear. However, further evaluation is needed. In humans, iatrogenic mechanisms for changes in oral pigmentation have been shown, such as implantation of dental amalgam causing a dark spot on the adjacent mucosa [9] and the disruption of normal melanogenesis within wounded skin causing dyspigmentation [10]. The published data are based on the use of different methodologies for evaluating and describing oral lesions. In order to make comparisons across different studies, it is necessary to develop a standard protocol for reproducible recording, description and evaluation of individual oral lesions. Standardisation will help governing bodies, veterinarians in practice, and researchers to compare findings in the field with the results of different studies. This information is needed as a basis to evaluate and interpret findings and to determine cause-and-effect relationships.

The aims of this study were to develop and apply a protocol for scoring findings around the lip commissures in horses, with particular focus on lesions caused by the bit. The objective evidence-based value of each parameter included in the protocol was evaluated by scoring and analysing the appearance of the oral commissures in 206 horses. The presence of natural pigmentation, potentially pathological pigment changes, interruption of the natural lining (scars), roughness, contusions/erosions, ulcers, and bleeding were identified from photographs of horses, including those that work regularly with a bit as well as horses that have never been bitted, to assess and understand the predictive value of the measured variables.

The specific goals were, first, to develop a protocol to examine the skin and mucosa at the commissures of the lips; second, to describe natural pigmentation of the skin and mucosa at the commissures of the lips; and third, to categorize and validate findings of the equine oral commissures in the context of pathological changes associated with the presence of a bit.

## 2. Materials and Methods

### 2.1. Study Design

**Figure 1:**
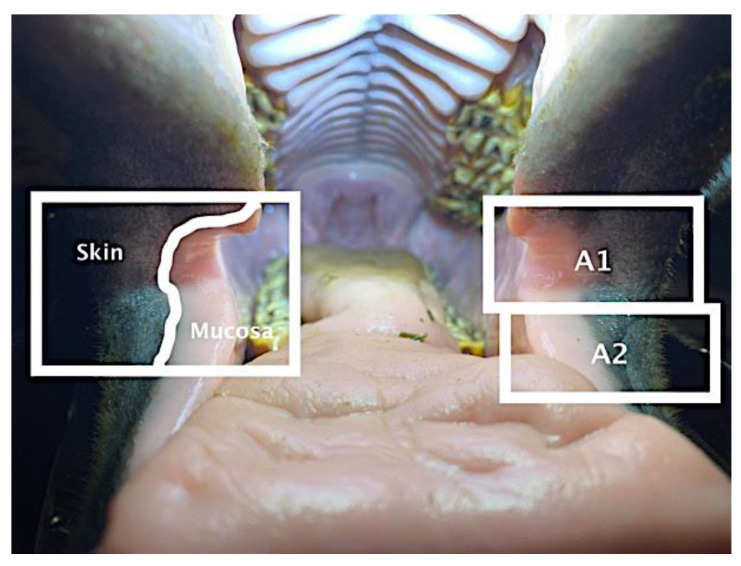
Open horse mouth showing area of inspection at the commissures of the lips. A1: area extending rostrally 0.5 cm from the commissure on the upper and lower lips, including areas of skin laterally and mucosa medially, both 1.5–3.0 cm wide. A2: area of the lower lip 0.5–2.0 cm rostral to the oral commissure, including areas of skin laterally and mucosa medially, both 1.5–3.0 cm wide. A3 (not labelled on photograph): upper and lower lips rostral to A1 and A2 on left and right sides.

The study was based on a convenience sample of 206 horses presented to 8 veterinarians for routine dental prophylaxis during January and February 2021. After sedation, the mouth was held open with a gag, while photographs were taken of the right and left oral commissures and adjacent areas of the lips, including both skin and mucosa (Figure 1). A questionnaire was completed by the veterinarian in collaboration with the owner, then signed by the owner. For each case, the date and examining veterinarian’s identification were recorded together with information describing the horse’s name, identification number, type (warmblood, Icelandic horse, pony), age, sex, colour, training level (none, low/medium, high), and discipline. The veterinarians recorded whether the horse was currently or had previously been bitted or whether it had never been bitted. They also recorded if there were markings in the haircoat, such as a snip or stripe, around the area of the oral commissures. The photographs and accompanying questionnaire were sent to one of the authors (MU).

### 2.2. Analysis of Photographs

The photographs were analysed by two of the authors (M.U. and L.B.), according to an Oral Commissure Assessment (OCA) protocol that was developed to describe the appearance of the oral commissures systematically and in standardized terms.

The anatomical location was coded 1–21 as described in Table 1 to distinguish between the involvement of skin, mucosa or both at each site. Note that the assigned numbers identify specific locations and do not indicate the severity of the findings. An example of the chart used to code the lesions can be found in Appendix A.

The category of natural pigmentation and the findings listed below were recorded for both the left and right commissures. Potentially pathological pigment changes include the following:

- Interruption of the natural lining (scars);
- Roughness;
- Contusion/erosion;
- Ulcer;
- Bleeding.

### 2.3. Description of Findings

**Figure 2:**
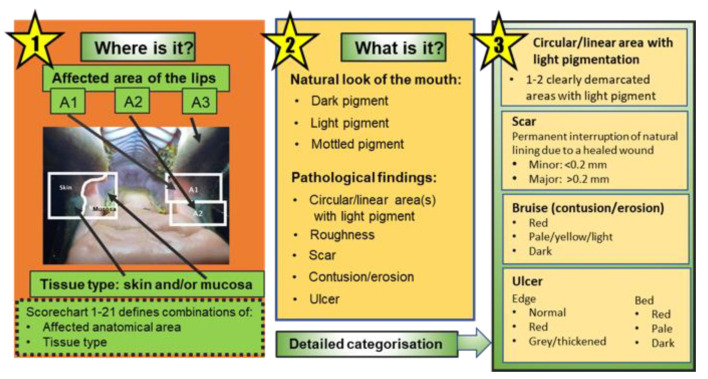
Diagram indicating the relationship between affected anatomical areas, the observed natural pigmentation of the mouth, and the types of potentially or definitely pathological findings recorded.

Figure 2 summarises the protocol including the affected areas and the descriptions of different types of potentially or definitely pathological findings.

#### 2.3.1. Pigmentation—Naturally Occurring

P1: Occurrence of overall light pigmentation

- Score 0: dark pigmentation;
- Score 1–21: anatomical location of light pigmentation;

P2: Irregular pigmentation, multiple areas of mottled pigmentation

- Score 0: no, irregular, or mottled pigmentation;
- Score 1–21: anatomical location/distribution of irregular or mottled pigmentation.

#### 2.3.2. Pigmentation—Potentially Pathological Pigment Changes

P3: One to two clearly demarcated circular areas of light to white pigmentation

- Score 0: none;
- Scores 1–21: anatomical location/distribution of circular areas of light pigmentation;

P4: One to two clearly demarcated circular areas of light grey pigmentation;

- Score 0: none;
- Scores 1–21: anatomical location/distribution of circular areas of grey pigmentation;

P5: One to two clearly demarcated linear areas of light to white pigmentation;

- Score 0: none;
- Scores 1–21: anatomical location/distribution of linear areas of light pigmentation;

P6: One to two clearly demarcated linear areas of light grey pigment;

- Score 0: none;
- Scores 1–21: anatomical location/distribution of linear areas of grey pigmentation.

#### 2.3.3. Changes in the Contours of the Mouth

S1: Healed minor wound (depth < 0.2 cm) with permanent interruption of natural lining

- Score 0: none;
- Scores 1–21: anatomical location/distribution of healed minor wounds;

S2: Healed major wound: Fissure (depth ≥ 0.2 cm) with permanent interruption of natural lining;

- Score 0: none;
- Scores 1–21: anatomical location/distribution of healed major wounds;

S3: Subjective evaluation of increased “roughness” of the skin/mucosa;

- Score 0: none;
- Scores 1–21: anatomical location/distribution of roughness.

#### 2.3.4. Contusion/Erosion (Bruises, Red Marks)

These lesions included contusions (intact skin, damaged capillaries causing red/brown bruising) or erosion (partial loss of epidermis):

CE1: Red—erythematous tissue;

- Score 0: none;
- Scores 1–21: anatomical location/distribution of red tissue;

CE2: Pale/yellow/light red—depigmentation/epithelialisation;

- Score 0: none;
- Scores 1–21: anatomical location/distribution of pale, yellow or light red tissue;

CE3: Dark—dark red/brown/black tissue;

- Score 0: none;
- Scores 1–21: anatomical location/distribution of dark red, brown or black tissue.

#### 2.3.5. Ulcer

These lesions exposed the dermis or underlying layers. For each lesion, there is a separate scoring for ulcer edge (UE) and ulcer bed (UB), where the ulcer edge is defined as the ~2 mm margin around the central ulcer bed.

UE1: Red erythematous tissue at the edge:

- Score 0: normal colour;
- Scores 1–21: anatomical location/distribution of red erythematous, tissue;

UE2: Greyish or thickened, unhealthy/fibrous tissue at the ulcer edge;

- Score 0: normal colour;
- Scores 1–21: anatomical location/distribution of greyish or thickened, unhealthy/fibrous tissue;

UB1: Red tissue—healthy red-pink tissue at the ulcer base:

- Score 0: none;
- Scores 1–21: anatomical location/distribution of healthy red-pink tissue;

UB2: Pale tissue—pale red/grey/white tissue at the ulcer base;

- Score 0: none;
- Scores 1–21: anatomical location/distribution of pale red/grey/white tissue;

UB3: Dark tissue—dark red/brown/black tissue at the ulcer base;

- Score 0: none;
- Scores 1–21: anatomical location/distribution of dark red/brown/black tissue.

#### 2.3.6. Bleeding

Bleeding is defined as the appearance of fresh blood in relation to a lesion at the oral commissure:

B1: Fresh blood related to a lesion;

- Score 0: none;
- Scores 1–21: anatomical location/distribution of bleeding.

### 2.4. Statistical Analysis

Recorded potential lesions were transformed into binary observations for statistical analyses. If the variable was coded with a number higher than zero, the finding was categorized as positive; if the variable was coded with 0, the finding was categorized as negative.

Bivariate analyses were performed on the associations between predictors and outcome variables, based on chi-square statistics (proc freq, SAS Institute). When cells had fewer than five observations, p-values were obtained from exact estimation. *P*-values for the effect of age were obtained from a logistic regression model with age as an explanatory variable to gain statistical power. *P*-values from the bivariate analysis are shown, and some analyses are supplemented with logistic regression for which *p* values are in bold.

If there were no statistically significant predictors for the lesion, confidence intervals were calculated for the prevalence (proc freq, SAS Institute). If more than one of the predictors were significant in bivariate analyses, a logistic regression (proc genmod, SAS Institute) model was performed, including the significant factors (*p* < 0.05). A backward selection process was performed, removing predictors that were non-significant. Interactions between the predictors were investigated and retained in the model if significant. If more than one predictor was significant, prevalence and confidence intervals were calculated, based on least square means (LSM) estimates. LSM generates prevalence and confidence intervals for an “average” horse, including the other predictors, for example, the effect of the training level for a horse of average age in the whole dataset.

## 3. Results

Probability values are shown in normal type for the results of the bivariate analyses and in bold type for the results of the logistic regressions.

### 3.1. Natural Pigmentation and Potentially Pathological Pigment Change

#### 3.1.1. Naturally Occurring Light or Mottled Pigmentation at the Commissures of the Lips (P1 and P2)

**Figure 3:**
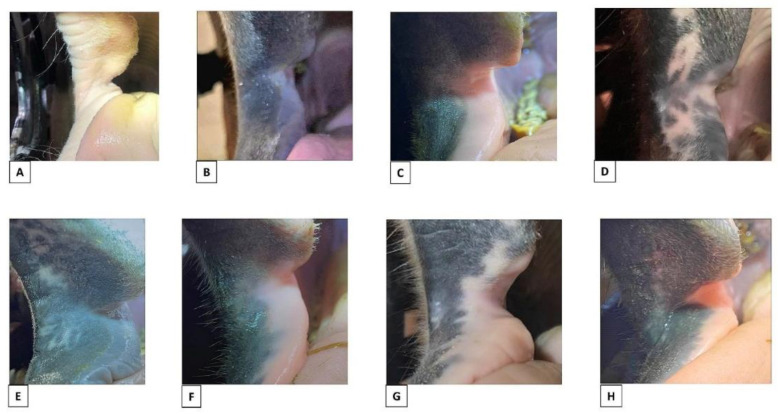
Examples of natural pigmentation colours and patterns. (**A**) Light with no mottled pigmentation. (**B**) Dark with no mottled pigmentation. (**C**) Light mucosa, dark skin, no mottled pigmentation. (**D**) Dark with mottled pigmentation of the skin and mucosa in area 1–3. (**E**) Dark with mottled pigmentation of the skin in area 1 + 3. (**F**) Light mucosa, dark skin, mottled pigmentation of mucosa area 2. (**G**) Light mucosa, dark skin, mottled pigmentation of mucosa and skin area 1–3. (**H**) Light mucosa, dark skin, mottled pigmentation of mucosa and skin area 1–3.

Naturally occurring light or mottled pigmentation at the commissures of the lips (P1 and P2) was present in 135/206 horses (Table 2, Figure 3).

The occurrence of overall light pigmentation (P1) or irregular/mottled pigmentation (P2) was common and in most cases present on both the left and right sides. Both P1 and P2 were more common in horses that have white markings, such as a blaze or snip, extending into the oral commissures, but the correlation did not reach statistical significance (*p* = 0.10).

The occurrence of natural light or mottled pigmentation was significantly associated with the type of horse, having a lower occurrence in taller horses (>148 cm at the withers) than in ponies and Icelandic horses (*p* = 0.02).

For discipline, a significant association was also shown to be caused by the group of trotters having a significantly lower frequency compared to other groups (*p* = 0.02). However, the validity of this result might be influenced by the small number of trotters included in the study (six horses) and the age/breed/type within this group being a confounding factor (Table 2).

Older horses were significantly more likely to have naturally occurring light or mottled pigmentation (P1–P2) than younger horses (*p* = 0.0001; *p* < 0.0001); the frequency was 44% in 0–4-year-olds, 48% in 5–9-year-olds, 73% in 10–14-year-olds and 94% in 15–20-year-olds. There was no correlation between naturally occurring light or mottled pigmentation and the colour of the horse’ body (*p* = 0.26), level of training (*p* = 0.24) or being currently or previously bitted (*p* = 0.49).

**Figure 4:**
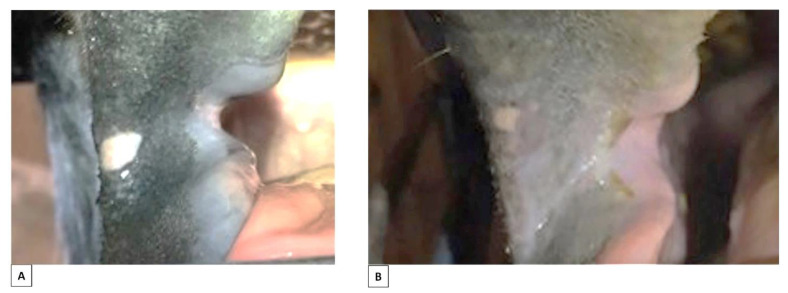
Natural pigmentation: spots of pigment potentially resembling pathological pigment changes from bit-induced lesions in horses that had never being bitted. (**A**) Slightly mottled dark mucosa, dark skin, no mottled pigmentation. A rare finding of a circular spot of clearly demarcated white pigment in area 1. (**B**) Light mucosa, dark skin, no mottled pigmentation. A rare finding of a circular spot of clearly demarcated white pigment in area 1.

Since there was no indication that naturally occurring light or mottled pigmentation (P1 and P2) was associated with the training of the horse or being currently or previously bitted (Figure 4), it is reasonable to classify P1 and P2 as naturally occurring pigmentation. However, it should be noted that P1, P2 and markings around the mouth can potentially be confounders to P3–P6.

#### 3.1.2. Naturally Occurring Light or Mottled Pigmentation (P1 and P2), Markings and Colour of the Horse Related to Potentially Pathological Pigment Changes (P3–P6)

There was a significant correlation between potentially pathological pigment changes (P3–P6) and the colour of the horse (*p* = 0.0004, ***p* =** **0.0003**) (Table 3), where the P3–P6 findings were more common in light-coloured horses than in those with a dark body colour (Table 2). There was no association between potentially pathological pigment changes from the bit (P3–P6), and naturally occurring light, mottled pigmentation (P1 and P2) (*p* = 0.15) or the presence of markings around the oral commissure (*p* = 0.88).

#### 3.1.3. Potentially Pathological Pigment Changes Related to Level of Training and Use of Horse (P3–P6)

**Figure 5:**
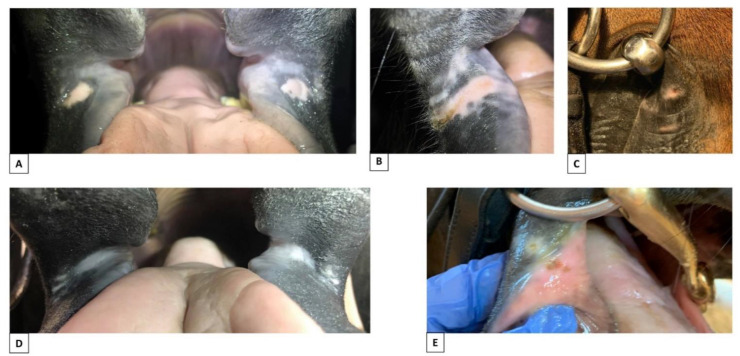
Potentially pathological pigment changes of the mouth of horses that had been bitted. (**A**) Circular spot on left and right side of the mouth, area 1–2, skin (horse known to have had lesions from the bit at the exact locations). (**B**) Linear area, area 2, skin + mucosa. (**C**) Circular spot, area 2, skin. (**D**) Linear grey area present on left and right side of the mouth, area 1, skin/mucosa. (**E**) Combined circular spot, skin + bruise, mucosa, area 2. The photos of bitted horses (**C**,**E**) are from horses that were not part of this study.

Potentially pathological pigment changes, P3–P6 findings, were present in 41/206 horses (Table 2, Figure 5).

In a 1:1 comparison between the never-bitted, reference group of horses with horses currently or previously bitted, there was no correlation with the P3–P6 findings (*p* = 0.20) (Table 3), although these findings were found in a higher percentage of currently or previously bitted horses than non-bitted horses (Table 2). Overall, 12% of horses in the non-bitted group were recorded with P3–P6 findings (Figure 4). Examples of lesions in bitted horses are shown in Figure 5.

The training level was significantly correlated with the occurrence of P3–P6 findings (*p* = 0.04; ***p* = 0.03**) (Table 3), with more findings in horses trained to a high level compared to those with medium- and low-level training (Table 2). The effect of discipline was not significant (*p* = 0.12). However, horses that were never bitted displayed a low level of potentially pathological pigment changes, as did the group of leisure horses (Table 2). The parameters of the training level and discipline are highly correlated in this data set.

P3–P6 potentially pathological pigment changes were more common in show jumping and dressage compared to no bit/untrained and leisure horses (Table 2) but were not correlated with age (*p* = 0.20) or type of horse (*p* = 0.06) (Table 3).

### 3.2. Changes in the Contours of the Mouth (S1–S2 and S3)

#### 3.2.1. Permanent Interruption of the Natural Lining (Scars), S1 and S2 Findings

**Figure 6:**
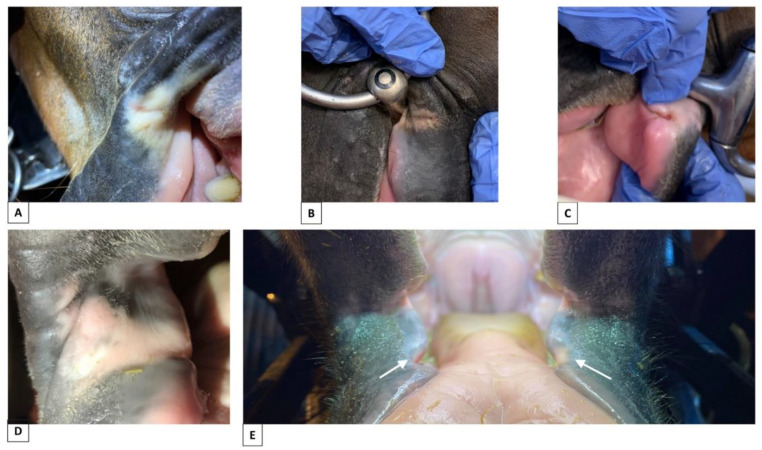
Permanent interruption of the natural lining (scars) due to previous lesions from the bit. (**A**) Minor scar in area 1, skin + fibrous scar tissue. (**B**) Minor scar in area 2, skin/mucosa + fibrous scar tissue. (**C**) Major fissure in area 2 skin/mucosa with ulcer at bottom. (**D**) Fissure in area 2, skin/mucosa + fibrous scar tissue. (**E**) Fissure in both left and right side, area 2, mucosa (white arrows). The photos of bitted horses (**B**,**C**) are from horses that were not part of this study.

Healed wounds with permanent interruption of the natural lining (scars, S1 and S2) were recorded in 7/206 horses, with bilateral scars in five horses. Anatomically, they were located in both A1 and A2, either in the mucosa or mucosa and skin (Figure 6).

Training level was significantly correlated with the S1 and S2 findings (*p* = 0.02, *p* = 0.03) (Table 3). In total, 18% of horses trained at high level presented with the S1 and S2 findings compared to 3% at mid-level and 0% in horses that were not being trained.

There was no correlation between permanent interruption of the natural lining (scars) and markings (*p* = 1), body colour (*p* = 0.84), discipline (*p* = 0.48), bit (*p* = 0.35), type of horse (*p* = 0.64) or age (*p* = 0.64) (Table 3). However, no scars were recorded in non-bitted horses (Table 2).

#### 3.2.2. Roughness, S3 Findings

**Figure 7:**
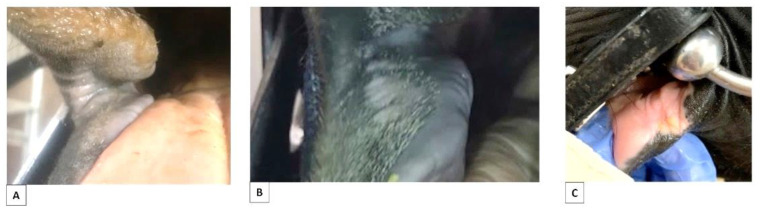
Examples of areas of roughness of the skin in horses with and without a history of being bitted. (**A**) Roughness in area 1, skin, of a horse being bitted. (**B**) Roughness in area 2, skin, of a horse never being bitted. (**C**) Roughness in area 2, skin, of a horse being bitted. The photo of a bitted horse (**C**) is from a horse was not part of this study.

Roughness was present in 8/206 horses (Table 2, Figure 7).

Roughness (S3) was not correlated to findings of scars (S1 and S2) (*p* = 1) or any other parameter: markings (*p* = 1), colour (*p* = 0.74), discipline (*p* = 0.50), training (*p* = 0.40), bit (*p* = 0.32), type of horse (*p* = 0.15) or age (*p* = 0.16) (Table 3). Roughness was not recorded for any high-level horse, and 7% of the S3 findings involved horses that were not bitted (Table 2).

### 3.3. Contusion/Erosion (CE1–3)

**Figure 8:**
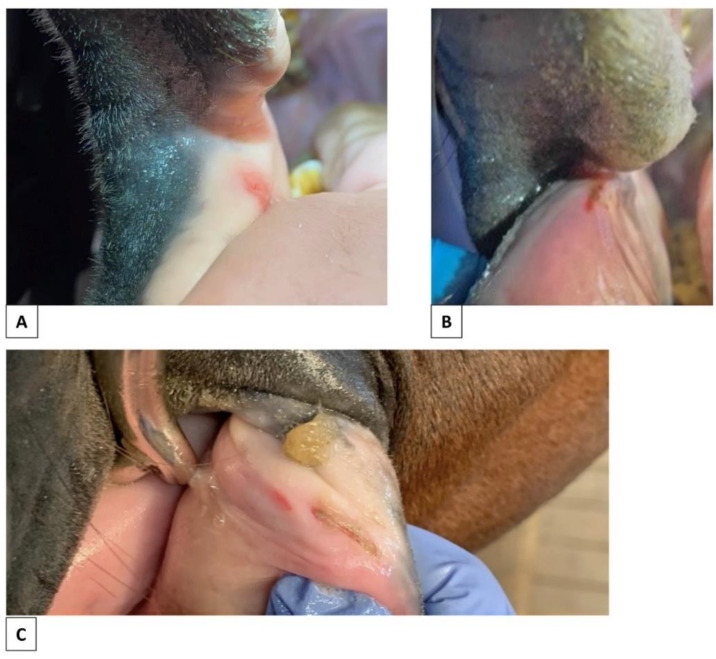
Examples of erosion/contusions (bruises). (**A**) Bruise area 2, mucosa. (**B**) Bruise area 2, mucosa. (**C**) Combined bruise (left) and ulcer (right), area 2, mucosa.

Only two horses (1 Icelandic, 1 pony) had a contusion or erosion, and both were classified as red erosions. One was not in training; one was trained at a low/intermediate level. Due to the low number of occurrences, no statistical analyses were performed (Figure 8).

### 3.4. Ulcer (UE1–2 and UB1–3)

Five horses had ulcers, and these were not correlated with any other parameter, which was likely due to the low numbers. Regarding level of training, one was recorded in a high-level horse, four at low/intermediate levels, and none in untrained or unbitted horses.

**Figure 9:**
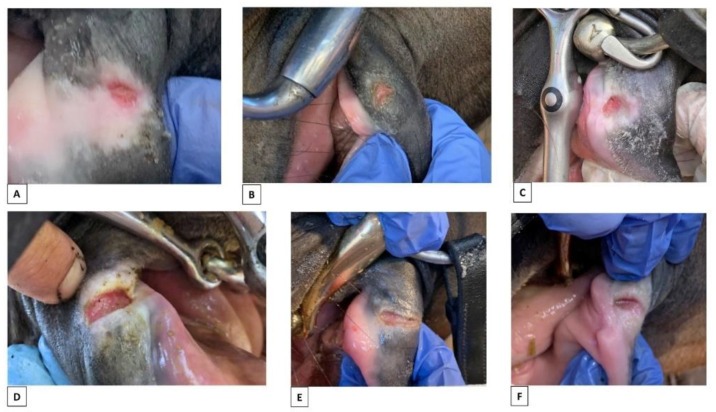
Ulcers in skin and mucosa of area 2 associated with use of a bit. (**A**) Ulcer exposed from the bottom of a fissure, area 2, skin/mucosa: normal edge, red bed + fibrous scar tissue. (**B**) Ulcer, area 2, skin: normal edge, red bed. (**C**) Ulcer, area 2, skin/mucosa: grey edge, red bed + fibrous scar tissue. (**D**) Ulcer, area 2, skin/mucosa: grey edge, red bed + fibrous scar tissue. (**E**) Ulcer, area 2, skin: grey edge, pale/yellow/light bed. (**F**) Ulcer exposed from the bottom of a fissure, area 2, skin/mucosa: grey edge, dark bed + fibrous scar tissue. The photos of bitted horses (**B**–**F**) are from horses that were not part of this study.

The ulcers were scored according to pathological appearance: one horse with a normal edge and red bed on one side; two horses with a red edge and red bed, one unilateral and one bilateral; one horse with a red edge and pale bed on both sides; and one horse with a red edge and dark bed on one side (Figure 9).

### 3.5. Bleeding

No horses were recorded with bleeding.

## 4. Discussion

This paper describes a consistent and thorough protocol for examining and describing pathological changes at and around the oral commissures. The OCA protocol can be used to document findings in relation to governance of horse welfare in sport. In this study, good visualisation was attained by sedating the horses and using a speculum for the examination. However, the protocol can equally be used for inspections on site at competitions without sedation or use of speculum. The structure of the protocol allows the user to focus only on pigmentation, scars or lesions if that is the scope of the current investigation; if the exact location of the findings is not of interest, the anatomical location score can be ignored, and observations assigned to just the left and right side. Some oral lesions can be seen as historical markers of previous bit use while others are acute and indicate recent or current use. More information is needed regarding the interpretation of what each lesion means in relation to equine management.

The availability of defined protocols and lesion classifications for evaluating and describing oral lesions in horses will help equestrian sport governing bodies, veterinary practitioners and researchers to report their findings in a standardized manner that facilitates making comparisons between different populations and studies. Validation of the predictive value and overall reliability of findings at both individual and population level is important to be able to use examination of the oral commissures of the horse as an evidence-based screening tool to evaluate bit-related problems and the associated welfare of the horse. The first step in the process is to define the range of natural appearances of the mouth since this has a bearing on how potentially pathological findings are interpreted.

In this study, it was shown that there is great variability in the natural appearance of the mouth, with overall light pigmentation or mottled, irregular pigmentation patterns being very common and usually bilateral. This type of pigmentation occurred more frequently in ponies and Icelandic horses than in horses >148 cm at the withers. This type of pigmentation also showed a statistically significant relationship to discipline as a consequence of 83% of trotters presenting with natural dark pigmentation of the commissures. Since there were only six trotters in the study, the link between pigmentation and discipline might not be valid. Pigmentation type was not associated with the training of the horse or being currently or previously bitted. Older horses were more likely to have naturally occurring light or mottled oral pigmentation than younger horses, with a gradual increase from 44% in 4-year-olds to 94% in 15–20-year-olds. Whether oral pigmentation changes during the life span of the horse needs further investigation.

Selection towards breeds with more or less pigment might occur incidentally during the selection of riding horses for performance in specific sports, and this could explain why the individual parameters can potentially act as confounders to each other. It can be concluded that a broad variability of the natural look of the mouth is apparent.

Potentially pathological pigment changes caused by the bit were recognized as clearly demarcated circular or linear areas of lighter pigment in the oral commissures and adjacent lips. This is consistent with findings of lighter pigment and scarring in humans caused by skin wounds. These changes had a higher incidence in lighter coloured horses, which might be due to lightly pigmented skin/mucosa being weaker and less resistant to wear than darker skin/mucosa. In people, black skin has greater cohesiveness than lighter coloured skin under some circumstances, but is also more likely to show mottled hyperpigmentation with an uneven skin tone [11]. Thus, dark skin might have a protective effect against the development of wear and pathological changes but can more easily display pigment changes. Additionally, it may be easier to recognise potentially pathological lightly pigmented areas on a dark background.

A higher number of currently or previously bitted horses had potentially pathological pigment changes, compared to non-bitted horses, but the difference did not reach statistical significance. A study described a significant association between ulcers and scarring/depigmentation at the oral commissures [8], indicating a close linkage between these two parameters and the seat of the bit. However, it is difficult to interpret the relative risks of body colour, naturally occurring pigment and other factors in relation to the findings of potentially pathological pigment changes in this data set. Therefore, we suggest that they be interpreted with caution at the individual level. In the non-bitted group, five horses had natural pigment spots similar to potentially pathological pigment changes, and this reinforces the need for caution in making any conclusions at individual level of the relationship between an apparent pigment change and the use of a bit.

Potentially pathological pigment changes were correlated to the horse’s level of training, with more findings in highly trained horses. This finding is consistent with other studies showing a higher frequency of lesions at the oral commissures in horses performing at higher levels [5,6,8]. Horses that had never been bitted showed a lower frequency of these changes.

As an overall conclusion, potentially pathological pigment changes (P3–P6) can serve as a warning of current or historically unsustainable use of the bit at a population level, which suggests that it may be a suitable screening method. Since these changes were occasionally recorded as an incidental finding in horses that were not trained or bitted, it is not a suitable parameter for unique identification of bit pathology in individual cases, due to its infrequent occurrence as a natural finding and the possibility that it is due to trauma unrelated to bit use.

Scars are chronic lesions that persist over time, and this cumulative effect likely explains their association with the training level being similar to the potentially pathological pigment changes caused by the bit and ulcers. This suggests that scars should be included as a marker of injury from the bit. Horses that had not been bitted had no scars, suggesting that this marker can be used to evaluate current and/or previous injury caused by the bit at an individual level, although with caution. It is also a valid indicator of current and/or previous bit problems at the population level.

Roughness of the skin/mucosa at the oral commissures does not appear to be a reliable parameter to evaluate findings caused by the bit, as there were no correlations to other parameters, including level of training in bitted and non-bitted horses. Thus, roughness around the commissures should be regarded as an incidental finding.

Contusion/erosions were recorded in only two horses, including one that was not trained or bitted. No conclusions can be drawn apart from the fact that the incidence is low and its validity as a marker at the individual and population levels is questionable, which is supported by another study in which more contusion/erosions were found, but the authors concluded that the relationship to bit use was inconsistent [8].

In the five ulcers reported here, the predominant features were that they had a red edge and red bed. In another study that used the same scoring system to evaluate 37 ulcers from high level horses, the edge distribution was normal in 22 horses and grey in 15 horses, and the bed was red in 24 horses, pale in 8 horses, and dark in 5 horses [8]. Similar to the study reported here, the ulcers predominantly had a red bed but there was more variation of the edge pathology. That study also reported a significant correlation between the presence of ulcers and scarring/pigment changes in the bit seat at the oral commissures [8].

A tendency for the frequency of commissural ulcers to be higher in ridden vs. unridden horses was reported [3], though the overall frequency of ulcers in that study was low. The frequency of ulcers was also low in our study with one occurrence in a horse at a high training level, four at low/intermediate training level, and none in the group of untrained horses that had not been bitted. From other studies, we have evidence of ulcers occurring primarily in horses competing at a high level in dressage [6], eventing [5], Icelandic competitions [7], trotting races [4], galloping races [2] and polo [2]. A low frequency of findings can be expected in a mixed population of horses primarily being trained at low to intermediate levels as in this study.

No horses were recorded with bleeding, which is consistent with the results from other studies [2,3,6,8] showing different frequencies of ulcers according to the population included but no bleeding. Although bleeding per se is not a reliable predicter of the presence of oral lesions from the bit, it should be included in a protocol for oral examination because the rare cases of bleeding from a wound in the oral region should always be addressed.

Pressure on the oral tissues from the bit is primarily due to rein tension, which is higher in canter than in trot or walk [12] and is particularly high during racing in which rein tension is twice as high as in riding horses [13]. It is reasonable to assume that the magnitude and duration of rein tension may have a causal relationship with the development of lesions.

Findings related to use of the bit can be divided into acute lesions, such as contusion/erosion or ulceration, and findings representing historical evidence of bit trauma, such as pigment changes or scars. In our cohort, the number of historical findings was far greater than the number of acute lesions. Based on the large increase in number and severity of oral lesions reported in Icelandic horses during the course of a competition [7], it would be useful to apply the protocol developed in this study to record lesions before and after training and competition in other sports.

Limitations of the study include the relatively small sample size, though the number was sufficient to test the overall application of the OCA protocol.

## 5. Conclusions

In conclusion, damage to the oral commissures may be due to pressure, friction, abrasion, iatrogenic causes or allergic reactions. The use of a protocol for evaluation of the oral commissures and adjacent areas proved useful when evaluating oral lesions in relation to bit use. The natural pigmentation patterns of the mouth varied and, on rare occasions, horses that had never been bitted presented with pigment spots resembling potentially pathological pigment changes associated with bit use. This makes it difficult to identify whether pigment changes are a consequence of bit use in an individual horse. Scars were reliable markers of previous problems with bit use at individual and population levels, but roughness was not. The validity of contusions and erosions being indicative of problems with the bit was questionable, because the numbers were too low for statistical analysis, and they were encountered in horses that were not being bitted. Ulcers were the key finding in relation to the assessment of current bitting problems. Their presence indicates current and previous discomfort and pain during the development of the lesion, which warrants further attention. Although ulcers were not associated with bleeding, it is recommended that bleeding should remain in the oral evaluation protocol because its presence always indicates a need for further investigation.

Validation of the predictive value and overall reliability of findings at both individual and population levels are needed before examination of the oral commissures can be used as an evidence-based screening tool to evaluate bit-related problems and equine welfare. The use of the OCA protocol in future studies will add to the significance of the findings in this study and, in the long term, assist with determining the distribution of natural pigmentation and pathological changes of the oral commissure in the horse population.

## Figures and Tables

**Table 1:** Score chart indicating anatomical location (A) and type of tissue (skin, mucosa, or both). Separate score charts were completed for natural pigmentation (P1–P2), potentially pathological pigment changes (P3–P6), interruption of the natural lining (S1–S2), roughness (S3), contusions/erosions (CE) and ulcers (U).

Area	Skin	Mucosa	Skin and Mucosa
A1	1	8	15
A2	2	9	16
A3	3	10	17
A1 + 2	4	11	18
A1 + 3	5	12	19
A2 + 3	6	13	20
A1 + 2 + 3	7	14	21

**Table 2:** Occurrence of natural pigmentation, potentially pathological pigment changes, interruption of natural lining/scars and roughness around the oral commissures according to the horse’s colour, discipline, level of training, being currently or previously bitted, type of horse and age.

Predictors	N	Naturally Occurring Light/Mottled Pigmentation (P1 and P2) Number (%)	Potentially Pathological Pigment Changes (P3 to P6) Number (%)	Interruption of Natural Lining (Scar) (S1 and S2) Number (%)	Roughness (S3) Number (%)
No marking	195	125 (64%)	39 (20%)	7 (3%)	8 (4%)
Marking around oral commissure	11	9 (91%)	2 (18%)	0	0
Dark colour body	150	95 (63%)	26 (17%)	6 (4%)	7 (5%)
Light colour body	32	25 (78%)	14 (44%)	1 (3%)	1 (3%)
Mix	24	15 (63%)	1 (4%)	0	0
Dressage	77	48 (62%)	16 (21%)	3 (4%)	4 (2%)
Event	2	2 (100%)	0	0	0
Jump	20	12 (60%)	7 (35%)	2 (10%)	0
Leisure	60	47 (78%)	10 (17%)	2 (3%)	1 (0.5%)
No bit/untrained	41	25 (61%)	5 (12%)	0	3 (1%)
Trotter	6	1 (17%)	3 (50%)	0	0
High training	11	5 (45%)	5 (45%)	2 (18%)	0
Low/Medium training	154	105 (68%)	31 (20%)	5 (3%)	5 (3%)
No training	41	25 (61%)	5 (12%)	0	3 (7%)
Bit	165	110 (67%)	36 (22%)	7 (4%)	5 (3%)
No bit	41	25 (61%)	5 (12%)	0	3 (7%)
Horse (>148 cm)	128	76 (59%)	27 (21%)	3 (2%)	5 (2%)
Icelandic	42	29 (69%)	6 (14%)	2 (5%)	0
Pony	36	30 (83%)	8 (22%)	2 (6%)	3 (1%)
0–4 years	32	14 (44%)	6 (19%)	0	3 (1%)
5–9 years	65	31 (48%)	17 (26%)	3 (5%)	1 (0.5%)
10–14 years	60	44 (73%)	10 (17%)	2 (3%)	4 (2%)
15–20 years	49	46 (94%)	8 (16%)	2 (4%)	0

**Table 3:** Results of bivariate analyses shown as *p*-values. Values in bold in parentheses are from logistic regressions.

Variables	Natural Pigmentation (P1 and P2)	Potentially Pathological Pigment Changes (P3 to P6)	Interruption of the Natural Lining (Scars) (S1 and S2)	Roughness (S3)
Marking around oral commissure	0.10	0.88	1	1
Colour of body	0.26	0.0004 **(0.0003)**	0.84	0.74
Discipline	0.02	0.12	0.48	0.50
Training	0.24	0.04 **(0.03)**	0.02 **(0.03)**	0.40
Bit	0.49	0.20	0.35	0.32
Type of horse	0.02	0.06	0.64	0.15
Age	0.0001 **(<0.0001)**	0.20	0.64	0.16

**Figure A1:**
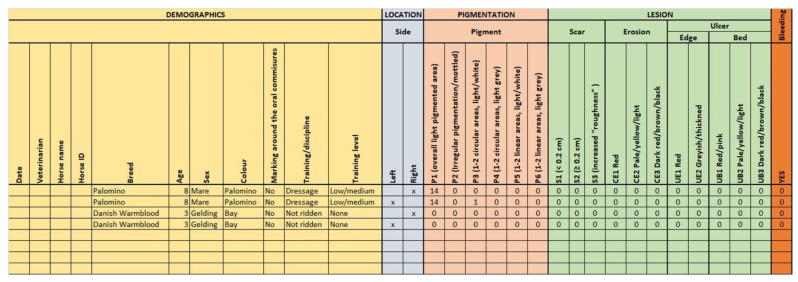
Example of the data recording sheet that includes demographics, oral location of lesions, and the presence of different types of pigmentation, lesions (scars, erosions, ulcers) and bleeding.

## Data Availability

Not applicable.
